# Oxidative Stress Aggravates Apoptosis of Nucleus Pulposus Cells through m^6^A Modification of MAT2A Pre-mRNA by METTL16

**DOI:** 10.1155/2022/4036274

**Published:** 2022-01-11

**Authors:** Peng-Bo Chen, Gui-Xun Shi, Tao Liu, Bo Li, Sheng-Dan Jiang, Xin-Feng Zheng, Lei-Sheng Jiang

**Affiliations:** ^1^Spine Center, Xinhua Hospital, Shanghai Jiaotong University School of Medicine, Shanghai 200092, China; ^2^Department of Orthopedic Surgery, Anting Hospital, Jiading District, Shanghai 200805, China

## Abstract

The process of intervertebral disc degeneration (IVDD) is complex, and its mechanism is considered multifactorial. Apoptosis of oxidative stressed nucleus pulposus cells (NPCs) should be a fundamental element in the pathogenesis of IVDD. In our pilot study, we found that the expression of MAT2A decreased, and METTL16 increased in the degenerative nucleus pulposus tissues. Previous studies have shown that the balance of splicing, maturation, and degradation of MAT2A pre-mRNA is regulated by METTL16 m^6^A modification. In the current study, we aimed to figure out whether this mechanism was involved in the aberrant apoptosis of NPCs and IVDD. Human NPCs were isolated and cultured under oxidative stress. An IVDD animal model was established. It showed that significantly higher METTL16 expression and lower MAT2A expression were seen in either the NPCs under oxidative stress or the degenerative discs of the animal model. MAT2A was inhibited with siRNA *in vitro* or cycloleucine *in vivo*. METTL16 was overexpressed with lentivirus *in vitro* or *in vivo*. Downregulation of MAT2A or upregulation of METTL16 aggravated nucleus pulposus cell apoptosis and disc disorganization. The balance of splicing, maturation, and degradation of MAT2A pre-mRNA was significantly inclined to degradation in the NPCs with the overexpression of METTL16. Increased apoptosis of NPCs under oxidative stress could be rescued by reducing the expression of METTL16 using siRNA with more maturation of MAT2A pre-mRNA. Collectively, oxidative stress aggravates apoptosis of NPCs through disrupting the balance of splicing, maturation, and degradation of MAT2A pre-mRNA, which is m^6^A modified by METTL16.

## 1. Introduction

As the main cause of low back pain, intervertebral disc degeneration (IVDD) imposes a great social and economic burden and leads to a poor life quality for sufferers [1–3]. The process of IVDD is complex, and its mechanism is considered multifactoral. It has been proved that abnormal apoptosis of the cells or disturbance of the extracellular matrix metabolism in the nucleus pulposus (NP) can lead to disc degeneration [4–7].

Oxidative stress is important in the pathogenesis of many diseases [8, 9]. In rats, the content of nitric oxide (NO, a marker of reactive oxygen species production) in degenerative intervertebral discs is higher than in healthy discs [10]. The percentage of nitrotyrosine-positive cells which are associated with oxidative stress in human NP increases with the progression of IVDD [11]. Oxidative stress has also been found to trigger autophagy, apoptosis, and aging of NPCs powerfully [12–14]. Thus, oxidative stress exists in intervertebral discs, and the apoptosis of NPCs induced by oxidative stress should be a fundamental element in the pathogenesis of IVDD. However, the exact mechanism of the apoptosis of NPCs under oxidative stress is not fully understood.

The human MAT2A synthesises S-adenosylmethionine (SAM) in all but liver cells, which has important functions in metabolism and epigenetics [15]. It has been reported that a mutation of MAT2A predisposes individuals to thoracic aortic aneurysms and reduces SAM in aortic smooth muscle cells, which in turn reduces glutathione activity and increases oxidative stress responses [16]. Inhibition of MAT2A function induces the expression of FasL, the formation of Fas-Disc and Caspase-8-dependent apoptosis in T cells [17]. SAM is able to ameliorate oxidative stress and lipid accumulation significantly in hepatic cells and in endothelial cells [18]. Li et al. reported that SAM was able to attenuate oxidative stress and neuroinflammation through glutathione metabolism modulation against amyloid-*β* [19]. It was also reported that SAM suppressed oxidative stress to reduce airway inflammation and fibrosis in a murine chronic severe asthma model [20]. Thus, there is a strong relationship between MAT2A-SAM and oxidative stress in tissues and cells.

In our pilot study, we found that the expression of MAT2A gene in nondegenerative healthy NP tissues (collected from young patients undergoing surgery for congenital hemivertebrae deformity) was higher than that in degenerative NP tissues (collected from adult patients undergoing surgery for degenerative lumbar disc diseases). However, the role of MAT2A in the pathogenesis of disc degeneration and in the apoptosis of NPCs under oxidative stress has never been investigated.

It has been reported that specific hairpin structures in MAT2A pre-mRNA are catalyzed by methyltransferase like 16 (METTL16) to produce m^6^A modification, which mediates the degradation of MAT2A pre-mRNA [21, 22]. In our pilot study, we also found that the METTL16 expression was much higher in the degenerative NP tissues than in the nondegenerative ones. Thus, we speculate that oxidative stress aggravates the apoptosis of the NPCs through m^6^A modification of MAT2A by METTL16 and finally leads to disc degeneration.

## 2. Materials and Methods

### 2.1. Clinical Tissue Samples

Experimental protocols involving human specimens and animals were approved by the Ethics Committee of Xinhua Hospital Affiliated to Shanghai Jiaotong University School of Medicine (XHECNSFC2021159). NP tissues from adult patients undergoing surgery for degenerative lumbar disc disease (3 male and 2 female patients, 35-68 years old, mean 56.4 years old. Pfirrmann level ≥ IV) were recognized as degenerative ones. NP tissues from young patients undergoing surgery for congenital hemivertebrae deformity (2 male patients and 1 female patient, 12-20 years old, mean 14.5 years old. Pfirrmann level ≤ II) were recognized as healthy and nondegenerative. Both degenerative and healthy NP tissues were performed immunofluorescence examination and quantitative real-time PCR (qRT-PCR). Healthy NP tissues were also used for cell isolation and culture.

### 2.2. Human Nucleus Pulposus Cell Isolation and Culture

After separated from patients intraoperatively, a part of healthy nondegenerative NP tissues was immediately stored in PBS and transported to a super clean bench. The NP tissue was then washed three times with aseptic PBS and isolated with collagenase type II (0.2%, Sigma-Aldrich) for 3-4 h at 37°C. The isolation solution contained 1% of a penicillin-streptomycin mix (Shanghai Yuanye Bio-Technology Co., Ltd.) and 0.1% of fetal bovine serum (Gibco, Thermo Fisher Scientific, Inc.). After isolation, the fragmented NP tissue was resuspended in DMEM/F12 combined with penicillin-streptomycin (1%) and fetal bovine serum (10%). Subsequently, NPCs would move out from the fragmented tissues after five days' culture at 37°C. We called it passage 0. When passage 0 cells were confluent to about 80%-90%, they were digested by Trypsin-EDTA for 3 minutes at 37°C. The digested cells were then resuspended and planted with a proper density into 25 cm^3^ culture dishes. The low passage (≤4) cells were used for the following experiments. There was no significant morphological change in NPCs between the initial (P1) and the following generations (P4). The primary NPCs were identified by characterization of their cell surface marker CD24 by flow cytometry [23].

### 2.3. NPCs with Oxidative Stress

In the current study, oxidative stress in NPCs was induced via the stimulation of tert-butyl hydroperoxide (TBHP). Previous studies indicated that TBHP increased the intracellular ROS level and apoptosis was involved in TBHP-induced human NP cell death [24, 25]. In our *in vitro* experiments, NPCs were exposed to 100 *μ*M TBHP when the cells grew to 60-80% cell density and cultured for 4 hours to increase the intracellular ROS level [26]. We examined the expression of METTL16 and MAT2A. We also used siRNA to reduce the expression of METTL16 and then stimulated the cells with 100 *μ*M TBHP for 4 hours to observe the change of apoptosis in the NPCs under this condition.

### 2.4. Lentivirus or siRNA Transfection in Human NPCs

To figure out the role of METTL16 in the apoptosis of NPCs, we added METTL16 overexpression lentivirus to the culture medium when the cells grew to 60-80% density. The lentiviruses used in cell experiments were purchased from Genomeditech (Shanghai, China). The titer of the virus was 1.13 × 10^8^ TU/ml, and it was diluted 1,000 times for infection. After 8 hours' culture, we replaced the medium. qRT-PCR and Western blotting were employed to determine the transfection efficiency. Human METTL16 sequence was used for cell experiments.

To figure out the effect of MAT2A on the apoptosis of NPCs, we applied siRNA. siRNA transfection was conducted using Rfect according to the manufacturer's instructions (Changzhou Bio-generating Biotechnologies Corp., China). The sequences of MAT2A siRNA (Genomeditech, China) were as follows: sense 5′-GGUUCGUGAAGCUGUUAAAtt-3′ and antisense 5′-UUUAACAGCUUCACGAACCtt-3′.

We also used siRNA to reduce the expression of METTL16 to observe the change of apoptosis in the NPCs under TBHP stimulation. The sequences of METTL16 siRNA (Genomeditech, China) were as follows: sense 5′-GGAUGCUCUUAAAGAAGAAtt-3′ and antisense 5′-UUCUUCUUUAAGAGCAUCCtt-3′. The negative control siRNA (NC-siRNA) does not target any human gene: 5′-UUCUCCGAACGUGUCACGUTT dTdT-3′ and antisense 5′-ACGUGACACGUUCGGAGAATT dTdT-3′.

### 2.5. RNA Extraction and qRT-PCR

Total RNA was extracted from NPCs and degenerative or nondegenerative disc tissues from operative patients using a TRIzol reagent (Beyotime, China). Transcriptor First Strand cDNA Synthesis Kit (Takara Biotechnology, Japan) was employed for reverse transcription. SYBR Green Kit Master Mix (Yeasen, China) was used for qRT-PCR. ABI 7500 Sequencing Detection System was employed to analyze the products according to the manufacturer's instructions. All human primers which have been reported used in the current study are listed in Table 1 [21]. We used GAPDH as an internal control.

### 2.6. Western Blotting

After discard culture medium, the cells were washed by PBS for three times. We then used the RIPA lysis buffer (Beyotime, China) to isolate total protein from cells. According to the protocol, protein samples were measured by the BCA kit (Beyotime, China) to determine the concentration and then separated by SDS-PAGE. Then, the separated proteins were transferred to polyvinylidene difluoride membranes waterishly. Membranes were incubated with nonfat milk (5%) for 1 h. Then, specific primary antibodies against METTL16 (1: 1000 dilution, ab186012), Bcl2 (1 : 1000 dilution, ab196495), cleaved Caspase3 (1 : 1000 dilution, ab13847) of Abcam (Cambridge, USA) and GAPDH (1 : 1000 dilution, 10494-1-AP), MAT2A (1 : 1000 dilution, 55309-1-AP), and Bax (1 : 1000 dilution, 50599-2-Ig) of Proteintech Group (Wuhan, China) were incubated overnight at 4°C with the membranes separately. We then incubated the membranes with horseradish peroxidase-conjugated anti-rabbit secondary antibody for 1 h after they were rinsed for three times. Finally, the membranes were visualized by the enhanced chemiluminescence detection system (Millipore, USA). Statistical images of WB were completed by the ImageJ software (USA), and the gray values of each protein relative to GAPDH were counted, *n* = 3.

### 2.7. Flow Cytometry Analysis for Apoptosis

Human NPCs were harvested after they were digested with 0.25% trypsin (Gibco, CA). We washed the NPCs with ice-cold PBS twice and resuspended them by 100 *μ*l binding buffer. Next, 5 *μ*l propidium solution and 5 *μ*l Annexin V-FITC solution (Vazyme, China) were added to the cell samples. After gently vortexed the samples and incubate them for 20 min in the dark at room temperature, we analyzed the samples by flow cytometry (Beckman CytoFLEX, Fullerton, CA) immediately.

### 2.8. ELISA

SAM is synthesized by MAT2A, and its concentration in the culture supernatant of NPCs was quantitatively determinated by S-Adenosylmethionine ELISA Kit (Biovision, USA). Centrifuge the supernatant for 20 minutes at 1000 × g, 4°C to remove insoluble impurities and cell debris. After saturation with PBS containing bovine serum albumin (5%), 96 well plates were successively incubated with the samples, 1 *μ*g/ml biotinylated biotin-detection antibody, and then with HRP-Streptavidin Conjugate working solution. Then, 90 *μ*l of TMB substrate was added into each well. We covered the plate and incubated it within 15-30 min at 37°C in the dark. Add 50 *μ*l of stop solution to each well and read the results at 450 nm within 20 minutes.

### 2.9. TUNEL Assay

A TUNEL apoptosis assay kit (Beyotime, China) was used to monitor the apoptosis of cells. After fixed the treated cells with 4% paraformaldehyde for 20 min at room temperature, 0.1% Triton X-100 (Beyotime, China) were used to permeabilize the NPCs for 5 min. Then, the cells were incubated with the reaction mixture of TUNEL at 37°C in the dark for 1 hour after washing in PBS. DAPI were used to stain the nuclei. Finally, we observed the apoptosis of cells by a fluorescence microscope and obtained images via Image Pro Plus version 6.0 (Media Cybernetics, USA). For each image, at least three views were chosen to analyze the rate of apoptosis via the ImageJ software version 1.48 (National Institutes of Health, USA). The number of cells that could be excited to emit red fluorescence (positive) and the total number of cells were counted from three fields, and the percentage of positive cells was calculated.

### 2.10. Immunofluorescence Examination of Tissues

Human healthy and degenerative NP tissues were fixed with 4% parafonnaldehyde. The samples were then dehydrated and cleared with dimethylbenzene. After embedded in paraffin, the samples were cut into 5 *μ*m thick serial slides. Sections were deparaffinized in xylene and rehydrated through graded ethanol. Then, they were immuno-stained after immersed in PBS two times for 5 min each. In order to retrieve the antigens, the sections were then immersed in 10 mmol/l citrate buffer for 10 min at 98°C and cooled down to room temperature. After that, sections were incubated in 5% bull serum albumin at room temperature for 30 min, followed by incubation with anti-METTL16 antibody (1 : 100 dilution, ab186012) overnight at 4°C. Next morning, the sections were washed and incubated with fluorescein isothiocyanate-conjugated secondary antibodies for 1 h at room temperature. Then, the sections were incubated in 5% rabbit IgG (Beyotime, China) for 1 hour at room temperature. Subsequently, we incubated the sections with anti-MAT2A antibody (1 : 100 dilution, ab207386) at room temperature for another 1 hour. Finally, the sections were labeled with DAPI for 5 min.

### 2.11. Immunofluorescence Examination of Cells

After removal of the culture medium, NPCs were washed with PBS for three times. 4% paraformaldehyde was used to fix the cells for 20 min, and 0.1% Triton X-100 were used to permeabilize the NPCs for 10 min. 5% bovine serum albumin blocked the cells for 1 h. Anti-METTL16 antibody (1 : 100 dilution, ab186012) incubated the cells in a humidified atmosphere at 4°C overnight. After washed with PBS for three times, NPCs were incubated with fluorescein isothiocyanate-conjugated secondary antibodies for 1 h at room temperature. Then, NPCs were incubated in 5% rabbit IgG (Beyotime, China) at room temperature for 1 hour and followed by incubation with anti-MAT2A antibody (Abcam, ab207386, 1 : 100 dilution) at room temperature for another 1 hour. Finally, the cells were labeled with DAPI for 5 min. We used fluorescence microscopy and Image Pro Plus version 6.0 (Media Cybernetics, USA) to observe the results and obtain images.

### 2.12. *In Vivo* Animal Studies

A disc degeneration animal model was established in 12-week-old mice (female, WT, C57BL/6) by needle puncturation method [24]. General anesthesia by intraperitoneal injection was administered using ketamine (100 mg/kg). Then, we made a sagittal small skin incision in the tail from Co6 to Co8 and punctured the Co7 coccygeal disc using a 23G syringe needle. The needle along the vertical direction of the tail was inserted into Co7 disc through the annulus fibrosus into the nucleus pulposus and then rotated in the axial by 180° to hold for 10 s. Successful disc degeneration was confirmed by X-ray examination and histomorphometric analysis 6 weeks later.

To figure out the role of METTL16 in disc degeneration, we injected METTL16 overexpression lentivirus into the Co7 discs of the mice. Six 12-week-old WT C57BL/6 mice were evenly distributed into the experimental group and the control group. Then, a total of 5 *μ*l solution containing METTL16 overexpression lentivirus or NC lentivirus was injected into the Co7 discs separately, with the injection depth about 1.5 mm using a 32G needle attached to a microsyringe. 32G needles have been shown not to cause disc degeneration [23]. The titer of the virus was 1.5 × 10^9^ TU/ml. The lentiviruses used *in vivo* were purchased from Genechem (Shanghai, China). Mouse METTL16 sequences were used in animal experiments. The injected mouse Co7 discs were examined by X-ray, histomorphometric, and immunohistochemical analysis 6 weeks later.

To figure out the role of MAT2A in disc degeneration, we injected the inhibitor of MAT2A, called cycloleucine (CLEU), into the Co7 discs of the mice. Another six 12-week-old WT C57BL/6 mice were evenly distributed into the experimental group and the control group. Then, a total of 5 *μ*l solution containing CLEU (400 *μ*m) or PBS was injected into the Co7 discs separately, with the injection depth about 1. 5 mm using a 32G needle attached to a microsyringe. The injected Co7 discs were examined by X-ray, histomorphometric, and immunohistochemical analysis 6 weeks later.

### 2.13. X-Ray Examination Method

Six weeks after disc injection, the tails of the mice were examined under radiography using Faxitron cabinet X-ray system. The height of the discs was observed to reflect the severity of IVDD.

### 2.14. Histomorphometric and Immunohistochemical Analysis of Mouse Discs

Human healthy and degenerative NP tissues were fixed with 4% parafonnaldehyde. The samples were then dehydrated and cleared with dimethylbenzene. After the samples were embedded in paraffin, we cut them into 5 *μ*m thick serial sections. Subsequently, the sections were deparaffinized in xylene and rehydrated through graded ethanol. A part of the sections were stained with Safranine O/fast green. The stained sections were then observed under a microscope; the number of cells and the structure of NP tissue were observed to evaluate the severity of IVDD. The degenerative degree of discs was evaluated by a histological grading scale [27–29]. The scale was based on five categories of disc changes: with 0 points for a normal disc and 15 points for a severely degenerated disc.

The other parts of the sections were immuno-stained. In order to retrieve the antigens, the sections were put in 10 mmol/l citrate buffer for 10 min at 98°C and cooled down to room temperature. Sections were incubated in 5% bull serum albumin at room temperature for 30 min and 1% HPz for 15 min to block the endogenous peroxidase activity. Then, we incubated the sections with anti-MAT2A antibody (Abcam, ab207386, 1 : 100 dilution) or anti-METTL16 antibody (Abcam, ab186012, 1 : 100 dilution) at 4°C overnight. After being washed thoroughly by PBS for three times, the sections were incubated with HRP-conjugated secondary antibody at room temperature for 30 min. Then, the sections were incubated with DAB peroxide substrate solution for 5-20 min. After rinsed in PBS, sections were counterstained with hematoxylin for 10 seconds. Then, the slides were mounted with neutral balsam after dehydrated in graded ethanol and cleared in xylene. We then observed the sections under a microscope to obtain the images via Image Pro Plus version 6.0 (Media Cybernetics, USA). Each image was chosen at least three views to analyze the expression of METTL16 or MAT2A via the ImageJ software version 1.48 (National Institutes of Health, USA). A uniform threshold value of brown area was set for the paired images, and the ratio of positive area to total area was calculated.

### 2.15. Statistical Analysis

Data were presented as means ± SD. The differences were analyzed by Student's *t*-test and one-way ANOVA using the GraphPad Prism software (Version 8.0). All experiments were performed at least three times separately. Differences were considered statistically significant at *p* < 0.05.

## 3. Results

### 3.1. Pilot Study

**Figure 1:**
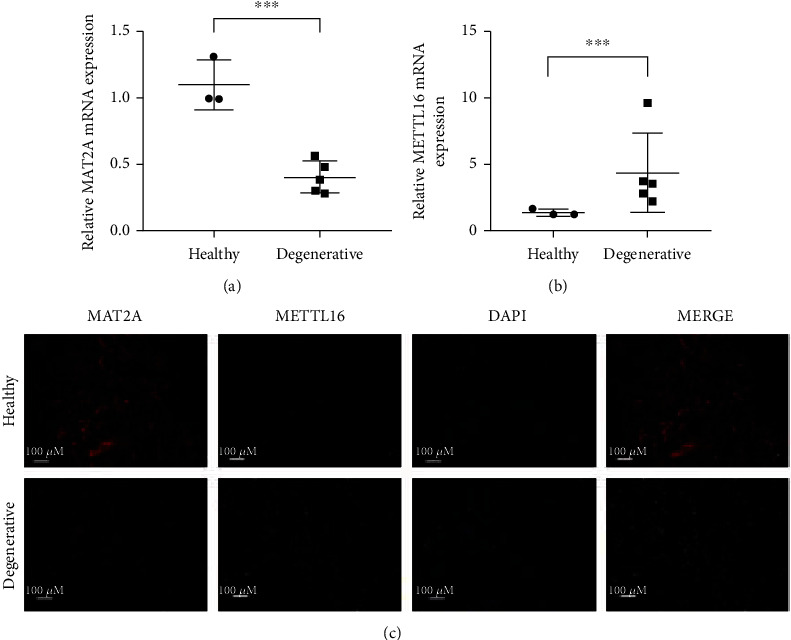
MAT2A decreased and METTL16 increased in human degenerative nucleus pulposus. (a) The expression of MAT2A in human NP tissues was analyzed by qRT-PCR. (b) The expression of METTL16 in human NP tissues analyzed by qRT-PCR. (c) Immunofluorescence assay for the expression of MAT2A and METTL16 proteins in human NP tissues. ^∗∗∗^*p* < 0.001.

To investigate the role of MAT2A in the pathogenesis of disc degeneration, we performed our pilot study. NP tissues were collected from healthy discs in young patients and from degenerative discs in adult patients as mentioned above. These tissues were subjected to PCR analysis and immunofluorescence assay. As expected, the mRNA expression of MAT2A in the degenerative NP was significantly lower than that in the nondegenerative NP (Figure 1(a)), while the mRNA expression of METTL16 was significantly higher in the degenerative NP than that in the healthy NP (Figure 1(b)). The immunofluorescent intensity for the protein expressions of MAT2A and METTL16 showed similar variation trends as compared with PCR analysis (Figure 1(c)).

### 3.2. Oxidative Stress Stimulates METTL16 Expression While Inhibits MAT2A Expression in Human NPCs

**Figure 2:**
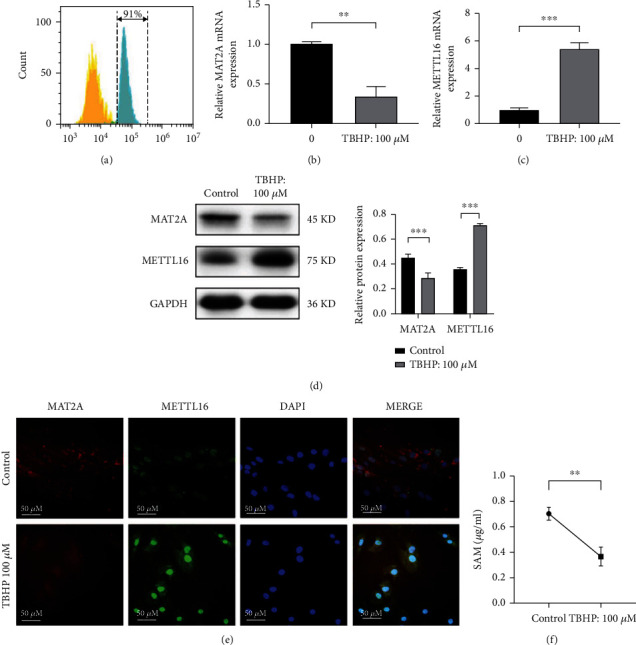
MAT2A was downregulated while METTL16 was upregulated when human nucleus pulposus cell was under oxidative stress. Human NPCs were exposed to TBHP (100 *μ*m, 4 hours). (a) NPC identification by characterization of their cell surface marker CD24 (b) The expression of MAT2A mRNA was detected by qRT-PCR. (c) The expression of METTL16 mRNA was detected by qRT-PCR. (d) Western blotting for the expression of MAT2A and METTL16 proteins. (e) Immunofluorescence assay for the expression of MAT2A and METTL16 proteins. (f) SAM concentration in the cell culture medium supernatant was detected by ELISA. *n* = 3 replicates per group, ^∗∗∗^*p* < 0.001, 0.001 ≤  ^∗∗^*p* < 0.05, ^∗^*p* < 0.05.

NPCs were successfully isolated from human nucleus pulposus tissue and identified by characterization of their cell surface marker CD24 by flow cytometry (Figure 2(a)). Human NPCs were stimulated with 100 *μ*m TBHP when the cells grew to 60-80% cell density. PCR demonstrated that the MAT2A mRNA expression decreased (Figure 2(b)) while the METTL16 mRNA expression increased (Figure 2(c)) in the cells under TBHP stimulation. Western blotting (Figure 2(d)) and immunofluorescence assay (Figure 2(e)) showed the same trends of these two proteins in the cells under TBHP stimulation as compared with PCR analysis. Because SAM is synthesized by MAT2A, its concentration evaluated by ELISA method was also significantly lower in the culture medium supernatant of the NPCs under TBHP stimulation (Figure 2(f)).

### 3.3. Downregulation of MAT2A or Upregulation of METTL16 in NPCs Promotes Apoptosis of the Cells

**Figure 3:**
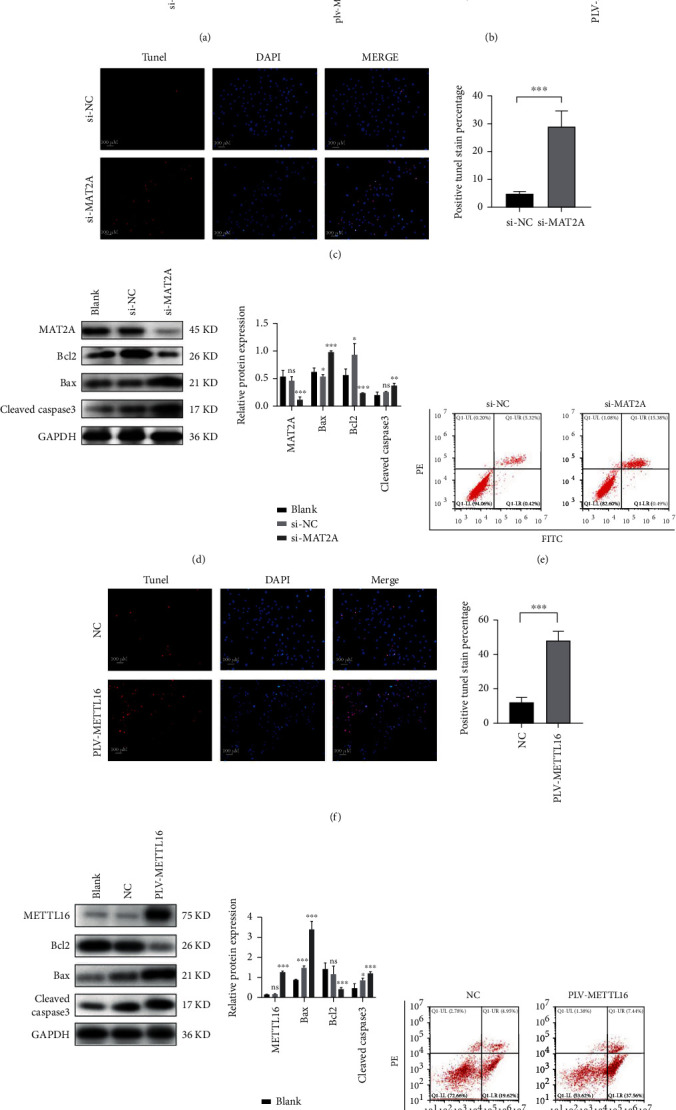
Downregulation of MAT2A or upregulation of METTL16 in NPCs promotes apoptosis. (a) Successful downregulation of MAT2A mRNA or upregulation of METTL16 mRNA in human NPCs confirmed by qRT-PCR. (b) SAM concentration in the supernatants of cell culture medium measured by ELISA method. (c) Apoptosis assayed by TUNEL staining significantly increased in the NPCs with MAT2A siRNA transfection. (d) Western blot analysis for the protein markers of apoptosis in cells with MAT2A siRNA transfection. (e) Flow cytometric analysis for the apoptosis of NPCs treated by MAT2A siRNA. (f) TUNEL staining for the apoptosis of NPCs with METTL16 overexpression. (g) The expression levels of Bcl2, Bax, and cleaved-Caspase3 detected by Western blot in cells with METTL16 overexpression. (h) Flow cytometric analysis of apoptosis in NPCs with METTL16 overexpression. *n* = 3 replicates per group, ^∗∗∗^*p* < 0.001, 0.001 ≤  ^∗∗^*p* < 0.05, ^∗^*p* < 0.05.

To investigate the function of MAT2A and METTL16 in the apoptosis of NPCs and finally their role in the pathogenesis of disc degeneration, we downregulated the expression of MAT2A in NPCs with small interfering RNA (siRNA) while upregulated the expression of METTL16 in the NPCs with lentivirus. Successful interference of MAT2A mRNA expression or overexpression of METTL16 mRNA was confirmed by PCR analysis (Figure 3(a)) in the cells. SAM concentration in the supernatant of cell culture medium was also significantly lower in the NPCs treated by MAT2A siRNA or METTL16 lentivirus in the cells (Figure 3(b)). With the decreased expression of MAT2A in the siRNA treated cells, the apoptosis of the cells significantly increased as evaluated by TUNEL staining (Figure 3(c)), Western blotting (Figure 3(d)), and flow cytometry (Figure 3(e)). Meanwhile, with the increased expression of METTL16 in the lentivirus treated cells, the apoptosis of the cells was also significantly increased (Figures 3(f)–3(h)).

### 3.4. The Effect of METTL16 on MAT2A

**Figure 4:**
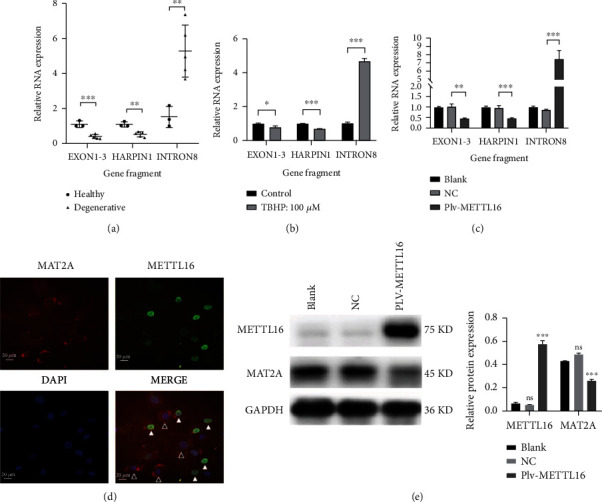
The effect of METTL16 on MAT2A. (a) In degenerative human NP tissues, qRT-PCR showed that exon1-3 and harpin1 reflecting the total of MAT2A pre-mRNA and MAT2A mRNA decreased, while intron8 reflecting MAT2A pre-mRNA increased. (b) In the NPCs stimulated by TBHP, qRT-PCR showed exon1-3 and harpin1 decreased while intron8 increased. (c) In the NPCs with METTL16 overexpression, qRT-PCR showed MAT2A exon1-3 and harpin1 decreased, while intron8 increased. (d) Immunofluorescence showed that the expression of MAT2A protein decreased when METTL16 was upregulated in NPCs. The expression of MAT2A was higher in the NPCs with unsuccessful overexpression of METTL16 (white arrow) than in the cells with successful overexpression of METTL16 (black arrow). (e) MAT2A and METTL16 proteins detected by Western blot in cells with METTL16 overexpression. *n* = 3 replicates per group, ^∗∗∗^*p* < 0.001, 0.001 ≤  ^∗∗^*p* < 0.05, ^∗^*p* < 0.05.

In our pilot study, we found that the expression of MAT2A in the degenerative NP was significantly lower, while the expression of METTL16 was significantly higher than that in the healthy nondegenerative NP. It has been reported that the balance of splicing, maturation, and degradation of MAT2A pre-mRNA is regulated by METTL16 protein [21, 22]. When METTL16 occupied its binding site on the UACAGAGA hairpin structure (adenosine underline) of MAT2A pre-mRNA, it promoted MAT2A pre-mRNA conversion to mature mRNA. However, when the UACAGAGA hairpin structure in MAT2A pre-mRNA was m^6^A modified by METTL16, MAT2A pre-mRNA was degraded. In the NP of the degenerative discs, we found that exon1-3 and harpin1, which reflect the total of MAT2A pre-mRNA and mRNA, decreased. Meanwhile, intron8, reflecting MAT2A pre-mRNA levels, increased (Figure 4(a)). In the NPCs under TBHP stimulation, the expressions of exon1-3 and harpin1 decreased, while intron8 expression increased (Figure 4(b)). Similar change patterns of exon1-3, harpin1, and intron8 were found in NPCs with overexpression of METTL16 (Figure 4(c)). These results suggested that m^6^A modification of MAT2A pre-mRNA by METTL16 should have an essential role in the mechanism of increased apoptosis of NPCs under oxidative stress and in the pathogenesis of disc degeneration.

Normally, when lentiviruses with puromycin resistance overexpress genes in cells, the unsuccessfully transfected cells will be killed by puromycin. Here, human NPCs transfected with overexpressing METTL16 lentivirus were not treated with puromycin. Thus, under immunofluorescence assay, there would be both successfully transfected cells and unsuccessfully transfected cells in one microscope view (Figure 4(d)). METTL16 was mainly expressed in the nucleus, while MAT2A was mainly expressed in the cytoplasm. As can be seen, the expression of MAT2A was less in the cells with successful overexpression of METTL16 (white arrow in Figure 4(d)) than in the cells without overexpression of METTL16 (black arrow in Figure 4(d)). The expression of MAT2A protein showed by Western blotting was also significantly lower in the cells with overexpression of METTL16 than in those without METTL16 overexpression (Figure 4(e)). These results confirmed that MAT2A could be regulated by METTL16 in the NPCs.

### 3.5. Increased Apoptosis of NPCs under Oxidative Stress Can Be Rescued by Downregulating METTL16 Expression in the Cells

**Figure 5:**
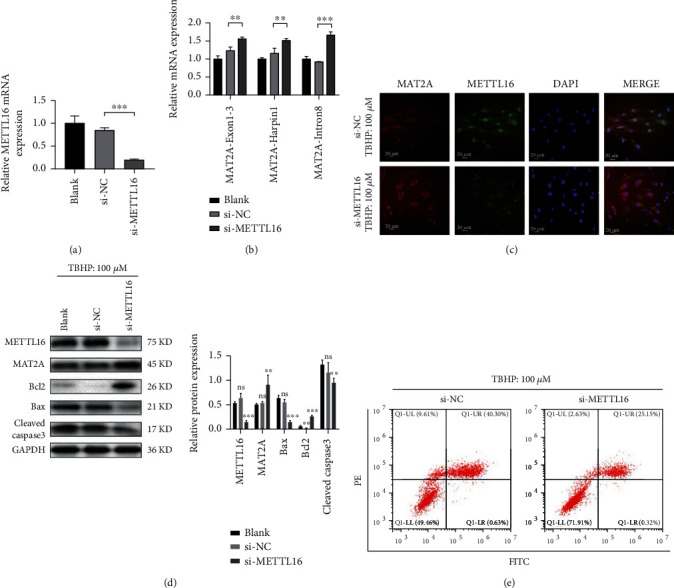
Increased apoptosis of human NPCs under oxidative stress can be rescued by reducing the expression of METTL16 in the cells. Human NPCs were transfected with METTL16 siRNA. (a) qRT-PCR analysis for transfection efficiency. (b) The cells were then stimulated with TBHP (100 *μ*m, 4 hours). And qRT-PCR was employed to detect different fragments of MAT2A pre-mRNA. (c) MAT2A protein detected by immunofluorescence. (d) The protein expression levels of METTL16, MAT2A, Bcl2, Bax, and cleaved Caspase3 were detected by Western blot in cells with METTL16 siRNA and under TBHP stimulation. (e) Flow cytometry for the apoptosis rate of the NPCs. *n* = 3 replicates per group, ^∗∗∗^*p* < 0.001, 0.001 ≤  ^∗∗^*p* < 0.05, ^∗^*p* < 0.05.

Small interfering RNA was used to downregulate the expression of METTL16 in the NPCs, and successful reduction was confirmed by PCR analysis (Figure 5(a)). As previously demonstrated, oxidative stress stimulated METTL16 expression while inhibiting MAT2A expression in human NPCs. Then, the cells with METTL16 siRNA were subjected to TBHP stimulation. We found that the amount of MAT2A exon1-3 and harpin1 mRNA in these cells was significantly higher than that in the cells without METTL16 siRNA, while intron8 expression increased at the same time (Figure 5(b)). These results confirmed that the balance of splicing, maturation, and degradation of MAT2A pre-mRNA is m^6^A modified by METTL16.

After TBHP stimulation, much more protein expression of MAT2A was found in the NPCs with METTL16 siRNA than in the cells without METTL16 siRNA, as evidenced by immunofluorescence assay (Figure 5(c)) and Western blotting (Figure 5(d)). Reducing the expression of METTL16 in NPCs substantially weakened the inhibition effect of oxidative stress on MAT2A expression in the cells.

Meanwhile, after TBHP stimulation, the apoptosis in the NPCs with METTL16 siRNA was significantly less than that in the cells without METTL16 siRNA, as evidenced by Western blotting (Figure 5(d)) and flow cytometric analysis (Figure 5(e)). Reducing the expression of METTL16 in the NPCs substantially rescued the apoptosis of the cells under oxidative stress.

### 3.6. *In Vivo* Animal Studies

**Figure 6:**
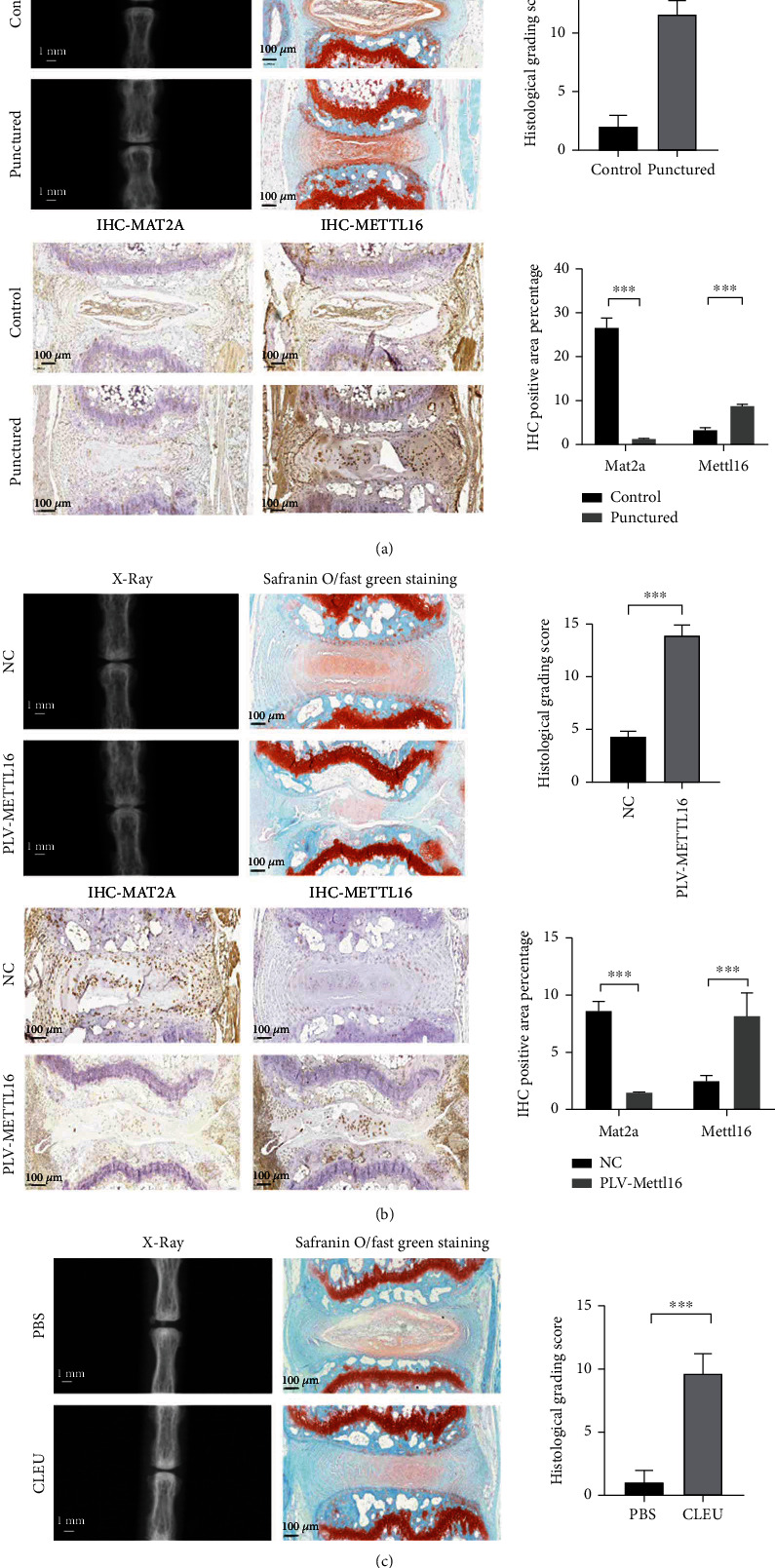
In vivo studies. (a) Verification of the disc degeneration animal model. X-ray examination revealed a significant decrease of the height of the punctured disc. Safranine O/fast green staining showed that the punctured NP tissue became more disorganized, and fewer cells could be seen. The histological grading score was significantly higher in the punctured disc. Immunohistochemical analysis demonstrated less MAT2A protein and more METTL16 protein levels in the punctured NP tissues. (b) Degenerative changes in the discs injected with METTL16 overexpression lentivirus. X-ray examination revealed significant decrease of the height of the discs. Safranine O/fast green staining showed that the NP tissue became more disorganized, and fewer cells could be seen. The histological grading score was also significantly higher. Immunohistochemical analysis demonstrated less MAT2A protein and more METTL16 protein levels in the NP tissues. (c) Degenerative changes in the discs injected with cycloleucine. X-ray examination revealed significant decrease of the height of the discs. Safranine O/fast green staining showed that the NP tissue became more disorganized, and fewer cells could be seen. The histological grading score was also significantly higher. *n* = 3 replicates per group, ^∗∗∗^*p* < 0.001, 0.001 ≤  ^∗∗^*p* < 0.05, ^∗^*p* < 0.05.

To support our speculation, we established a mouse model of disc degeneration by needle puncturation in the caudal intervertebral discs. Six weeks later, successful establishment of the model was verified. The height of the punctured intervertebral disc significantly decreased under X-ray examination. Safranine O/fast green staining demonstrated that the punctured NP tissue became more disorganized, and fewer cells were seen in it. The histological grading score was significantly higher in the punctured disc (Figure 6(a)). In accordance with our pilot study, the expression of MAT2A decreased while the METTL16 expression increased in the punctured NP tissues (Figure 6(a)).

To support our *in vitro* findings, we injected METTL16 lentivirus into the discs to increase the expression of METTL16 in the NP tissues. Six weeks later, we performed X-ray and histological examinations on the discs. The height of the intervertebral discs with METTL16 lentivirus injection became smaller. Safranine O/fast green showed that the NP with METTL16 lentivirus injection became more disorganized, and fewer cells could be seen in it. The histological grading score was also significantly higher (Figure 6(b)). Meanwhile, the expression of MAT2A protein significantly decreased in the NP tissues with METTL16 lentivirus injection (Figure 6(b)). These results indicated that increasing the expression of METTL16 in NP tissues could decrease MAT2A expression and aggravate disc degeneration *in vivo.*

To confirm that functional inhibition of MAT2A in NP will increase the apoptosis of NPCs and lead to disc degeneration, we injected the inhibitor of MAT2A called cycloleucine (CLEU) and the control substance phosphate buffered saline (PBS) into the caudal intervertebral discs. Compared with the PBS group, the height of the intervertebral discs in the CLEU group was smaller (Figure 6(c)), and the NP became more disorganized, and fewer cells could be seen in it. The histological grading score was also significantly higher (Figure 6(c)).

## 4. Discussion

m^6^A modification has been considered as a common RNA modification mode for decades [30, 31], which is a reversible process, including methylation and demethylation. In addition, there are m^6^A recognition enzymes, which perform biological functions by recognizing m^6^A sites on RNA to increase translation or promote degradation. Previous studies have shown that the balance of splicing, maturation, and degradation of MAT2A pre-mRNA is regulated by METTL16 protein [21, 22]. In the current study, we found that this mechanism was involved in the apoptosis of NPCs and IVDD.

Although the mechanism of intervertebral disc degenerative disease is not fully understood, aberrant apoptosis of NPCs has been recognized to have an important role in its development [32–35]. Proteoglycan and type II collagen produced by NP are the main molecules needed for maintaining the gelatinous properties of NP tissues [4, 35–37]. Abnormal apoptosis may lead to the disorder of extracellular matrix metabolism. Cumulative studies have shown that interventions targeting nucleus pulpocytes apoptosis can alleviate the disorder of extracellular matrix metabolism and even slow down the progression of IVDD [35, 38–43]. Therefore, we focus on NP cell apoptosis and target at its interventions to improve our understanding on the pathogenesis of IVDD and provide potential therapeutic strategies.

Multiple lines of evidence have shown that oxidative stress is involved in the development and progression of IVDD and plays roles in regulating the vitality and function of NPCs. The imbalance between ROS and endogenous antioxidant levels can lead to oxidative stress and, worse, apoptosis through mitochondrial dependent and mitochondrial independent pathways [44]. Redox-sensitive apoptosis signal–regulating kinase 1 (ASK1) signalosome and its downstream JNK pathway have been emphasized. However, in recent years, oxidative stress was found to be closely related to SAM. As a high-energy methyl donor for DNA, RNA, and proteins, SAM is necessary for most methylation events and is of great significance for gene regulation [45–47]. The methylation of DNA and histones is related to the stability and expression of genome. Methyltransferase inhibition or SAM decrease can cause different kinds of cells to undergo apoptosis through a diversity mechanisms [48–51]. SAM reduces airway inflammation and fibrosis by inhibiting oxidative stress in mice with chronic severe asthma [20]. SAM alleviates fatty acid-induced steatosis and oxidative stress in liver and endothelial cells [18]. Thus, there is a strong relationship among apoptosis, oxidative stress, and SAM.

In this study, we found that SAM decreased significantly in the culture medium supernatant of human NPCs under TBHP stimulation. MAT2A, the producer of SAM, also decreased significantly in the NPCs under oxidative stress and in the human degenerative NP tissues. In the disc degeneration animal model, decreased MAT2A expression was also found in degenerative NP tissue tissues.

We then investigated the role of MAT2A or SAM in NP cell apoptosis and the development of IVDD. We applied siRNA to reduce the expression of MAT2A in the NPCs. With the decreased MAT2A expression, the level of SAM in the supernatant of the cells was significantly reduced, while the apoptosis of NPCs significantly increased. Our *in vivo* study also demonstrated that when the function of MAT2A was inhibited, more serious degeneration of the caudal intervertebral discs in mice could be seen.

METTL16 has been reported to catalyze specific hairpin structures in MAT2A pre-mRNA to produce m^6^A modification, which mediates the degradation of MAT2A pre-mRNA [21, 22]. METTL16 can also occupy the specific hairpin structure of MAT2A pre-mRNA without m^6^A modification and promote its conversion to mature mRNA [21, 22, 52]. In our pilot study, we found METTL16 increased in the NP tissue of the degenerative discs. METTL16 was also found increased significantly in the NPCs under oxidative stress. In the needle puncture IVDD model, increased METTL16 was also confirmed in the NP tissue of the degenerative discs. Thus, we suspected that the increased METTL16 should be responsible for the decrease of MAT2A in the circumstances mentioned above.

In the NP tissues of the degenerative discs and NPCs under TBHP stimulation, we found that exon1-3 and harpin1, which reflect the total of MAT2A pre-mRNA and mRNA, decreased. Meanwhile, intron8, reflecting MAT2A pre-mRNA levels, increased. Our *in vitro* study of NPCs with overexpression of METTL16 showed similar change patterns of exon1-3, harpin1, and intron8. These results strongly implied that in the degenerative discs and in the NPCs under oxidative stress, the decreased expression of MAT2A mRNA was a result of the degradation of the MAT2A pre-mRNA. The balance of splicing, maturation, and degradation of MAT2A pre-mRNA is m^6^A modified by METTL16.

Decreased expression of MAT2A mRNA will lead to the shortage of MAT2A protein and SAM and aberrant apoptosis. Our *in vivo* study confirmed that in the NP tissues, overexpression of METTL16 could cause the decrease of MAT2A and the increase of apoptosis. Meanwhile, our *in vitro* study showed that the increased apoptosis of NPCs under oxidative stress could be rescued by downregulating METTL16 expression in the cells.

Therefore, we can conclude that when the intervertebral disc NP is in the state of oxidative stress, the expression of METTL16 protein will increase for some unknown reasons, which will lead to the imbalance of MAT2A pre-mRNA degradation and maturation. Then, the MAT2A mRNA and protein decrease, which will lead to low SAM level. Ultimately, this will cause apoptosis of NPCs and lead to disc degeneration. This study revealed a unique pathway of apoptosis in NPCs under oxidative stress, namely, the imbalance between maturation and degradation of MAT2A pre-mRNA, which is m^6^A modified by METTL16.

However, there are several limitations of this study. The reason that METTL16 increased in the NPCs under TBHP stimulation and in the development of IVDD was still unclear. How the situation of low SAM levels causes NP cell apoptosis needs to be explored.

## 5. Conclusions

Oxidative stress aggravates apoptosis of NPCs through disrupting the balance of splicing, maturation, and degradation of MAT2A pre-mRNA, which is m^6^A modified by METTL16.

## Figures and Tables

**Table 1:** Human primer sequences.

Primer name	Primer sequences (5′ to 3′)
MAT2A exon 1-3 F	CCACCCAGATAAGATTTGTGACC
MAT2A exon 1-3 R	GATGTAATTTCCCCAGCAAGAAG
MAT2A intron 8 F	AAGTGGGTTGCTCAAGGTTT
MAT2A intron 8 R	CCTGGCTCAACAAATACGAA
MAT2A hairpin 1 F	CATGGGAAGTGCCCAAAAAG
MAT2A hairpin 1 R	CAGAGCTTGAAGGCTTCTCT
METTL16 F	ACAGAAGACACTCCTGATGG
METTL16 R	TTAACAGAACTAGGCGGAGG
GAPDH F	AGCCTCAAGATCATCAGCAATG
GAPDH R	ATGGACTGTGGTCATGAGTCCTT

## Data Availability

The datasets used and/or analyzed during the current study are available from the corresponding author on reasonable request.
